# Regulatory RNAs: A Universal Language for Inter-Domain Communication

**DOI:** 10.3390/ijms21238919

**Published:** 2020-11-24

**Authors:** Emma Layton, Anna-Marie Fairhurst, Sam Griffiths-Jones, Richard K. Grencis, Ian S. Roberts

**Affiliations:** 1 Lydia Becker Institute of Immunology and Inflammation, Division of Infection, Immunity and Respiratory Medicine, School of Biological Sciences, Faculty of Biology Medicine and Health, University of Manchester, Manchester M13 9PT, UK; emma.layton@postgrad.manchester.ac.uk (E.L.); Sam.Griffiths-Jones@manchester.ac.uk (S.G.-J.); 2 Institute of Molecular and Cell Biology, A*STAR, 61 Biopolis Drive, Singapore 138673, Singapore; annamarie@imcb.a-star.edu.sg; 3 Division of Evolution and Genomic Sciences, School of Biological Sciences, Faculty of Biology Medicine and Health, University of Manchester, Manchester M13 9PT, UK

**Keywords:** RNA, miRNA, microbiota, communication, extracellular vesicles

## Abstract

In eukaryotes, microRNAs (miRNAs) have roles in development, homeostasis, disease and the immune response. Recent work has shown that plant and mammalian miRNAs also mediate cross-kingdom and cross-domain communications. However, these studies remain controversial and are lacking critical mechanistic explanations. Bacteria do not produce miRNAs themselves, and therefore it is unclear how these eukaryotic RNA molecules could function in the bacterial recipient. In this review, we compare and contrast the biogenesis and functions of regulatory RNAs in eukaryotes and bacteria. As a result, we discovered several conserved features and homologous components in these distinct pathways. These findings enabled us to propose novel mechanisms to explain how eukaryotic miRNAs could function in bacteria. Further understanding in this area is necessary to validate the findings of existing studies and could facilitate the use of miRNAs as novel tools for the directed remodelling of the human microbiota.

## 1. Regulatory RNAs in Eukaryotes

The expansive non-coding regions of eukaryotic genomes are now known to encode many important regulatory elements such as promoters, enhancers and long non-coding RNAs (lncRNAs) that influence the transcription of both protein-coding and non-coding regions [1,2]. Some regions function as post-transcriptional gene expression regulators, such as microRNAs (miRNAs), small interfering RNAs (siRNAs) and P-element-induced wimpy testis (PIWI)-interacting RNAs (piRNAs) that bind to their targets through complementary base pairing (Table 1).

In eukaryotes, miRNAs and siRNAs are derived from longer precursor molecules and processed into smaller molecules of approximately 21 to 25 nucleotides in length [3]. Both miRNA and siRNA mediate post-transcriptional gene silencing through the RNA-induced silencing (RISC) complex and the AGO subfamily of Argonaute proteins. The key difference is that miRNAs can bind to targets with imperfect complementarity, enabling one miRNA to target hundreds of messenger RNAs (mRNAs), whereas siRNAs generally require perfect complementarity to their target sequence [4]. Additionally, the siRNA silencing mechanism can be amplified by the action of RNA-dependent RNA polymerases (RdRPs). These enzymes produce secondary siRNAs by using the target mRNA as a template [5,6]. However, it is unclear whether such amplification occurs in vertebrates, as they do not possess RdRP genes. miRNAs and siRNAs associated with AGO and RISC can act in the defence against exogenous viruses by targeting key viral genes [7,8]. Small RNAs also prevent transposon transcription through the direction of epigenetic modifications and heterochromatin formation [9,10]. In particular, the piRNA family of small non-coding RNAs, are a primary form of defence against transposons and are also critical for fertility in animals [10,11]. piRNAs mediate transcriptional silencing through the PIWI subfamily of Argonaute proteins. Silencing occurs via the ping-pong cycle, during which a piRNA-bound transposon is cleaved by PIWI, generating another piRNA of the opposite orientation [12]. piRNAs are thought to mediate transposon silencing through heterochromatin formation and *de novo* DNA methylation, although the precise mechanisms through which this occurs are not yet clear [13]. These classes of regulatory RNA are each reminiscent of the distinct classes of bacterial regulatory RNAs. However, evidence suggests that miRNAs specifically are the key mediators of inter-domain communications [14,15,16].

### 1.1. miRNA Biogenesis

**Figure 1:**
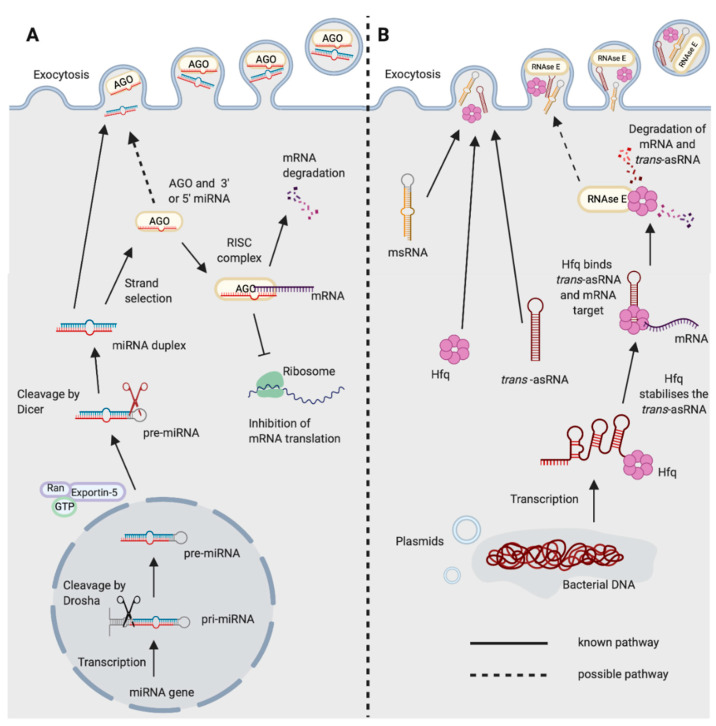
Biogenesis and functions of (**A**) miRNAs in eukaryotes and (**B**) *trans*-asRNAs in bacteria. (**A**) Eukaryotic miRNAs are transcribed in the nucleus into a pre-cursor primary-miRNA (pri-miRNA) transcript. The pri-miRNA is cleaved by Drosha (black scissors) to form a shorter hairpin, the pre-miRNA. The pre-miRNA is exported from the nucleus by exportin-5 in conjunction with ran-GTP. In the cytoplasm, the pre-miRNA is cleaved further by Dicer (red scissors) to form a miRNA duplex. One strand from the miRNA duplex is preferentially loaded onto Argonaute (AGO) forming the RNA-induced Silencing Complex (RISC). The miRNA of the RISC complex facilitates binding to the target mRNA (requiring only partial complementarity). Catalytically active AGOs (AGO2 in humans) can cleave and degrade the mRNA target. The RISC complex can also inhibit ribosomal translation of mRNAs. Exocytosis of miRNAs in Extracellular Vesicles (EVs) has been shown in eukaryotes [14]. It is not yet known whether the miRNA could be loaded onto AGO prior to exocytosis. (**B**) Newly transcribed bacterial *trans*-asRNAs can be stabilised by Hfq. Hfq can also facilitate binding between the *trans-*asRNA and the mRNA target [29,30]. In Gram-negative bacteria, Hfq can facilitate binding of the *trans-*asRNA and mRNA to RNase E which degrades both the asRNA and mRNA [31]. It is unclear whether RNase E can be exported from bacteria, but it does possess a domain capable of binding the membrane of phospholipid vesicles in vitro [32]. Bacteria have been shown to export microRNA-size RNA (msRNA) and *trans*-asRNAs in EVs [33,34,35]. Hfq can also be exported from bacteria in EVs [36] (Created with BioRender.com).

miRNAs were first discovered in 1993 in *Caenorhabditis elegans* [17]. They are post-transcriptional gene regulators, typically 21–25 nucleotides in length, and are found in almost all plants and animals [18,19,20]. They are transcribed in the nucleus by RNA polymerase II to form a long pre-cursor primary miRNA molecule which creates a hairpin structure (Figure 1A) [21]. In animals, the hairpin is excised by the endonuclease Drosha and a shorter pre-miRNA hairpin molecule forms which is exported into the cytoplasm by exportin-5 [22,23]. The endonuclease Dicer then cleaves the terminal hairpin leaving an RNA duplex of approximately 22 nucleotides in length. Typically, one of these strands is preferentially loaded onto the RISC complex, directing the enzymatic complex to the target mRNA [24]. The RISC complex consists of the miRNA and an Argonaute protein, although there can also be many other binding partners [25]. The complex can either degrade the target or inhibit its translation, dependent on the level of complementarity [26]. miRNAs can additionally up-regulate the expression of the target mRNA, although this is less common [27]. miRNAs typically bind to the 3′ Untranslated Region (UTR) of the target mRNA with incomplete base pairing, often only requiring complementarity to the “seed sequence” of the miRNA, at nucleotides 2–8 [28]. This enables a single miRNA to have many target transcripts.

### 1.2. miRNAs in Disease

Currently, there are 1917 identified miRNA loci in the human genome, with 60% of human protein-coding genes estimated to be regulated by at least one miRNA [37,38]. They form complex signalling networks which regulate important biological processes such as cell differentiation and proliferation, apoptosis, metabolism, the immune response, development and ageing [39,40,41,42,43]. Due to the critical roles of miRNAs in such a range of biological processes, they are also ideal targets for pathogens that need to alter their environment to establish infection. Host miRNAs play a fundamental role in shaping the innate and adaptive immune responses, and can also directly target pathogens and inhibit their replication [44,45,46]. Conversely, pathogens can also produce miRNAs to disrupt the immune response, facilitating their own survival [7,47]. Human Immunodeficiency Virus 1 (HIV-1) encodes a pre-miRNA sequence in its genome (HIV1-miR-H1). HIV-1 exploits the human host’s miRNA processing machinery to convert this into a functional mature miRNA. This has critical roles in HIV-1 pathogenesis due to its repression of host anti-viral responses, including the host anti-viral miRNA, hsa-miR-149 [48,49,50]. Other viruses capable of producing miRNAs include Herpes Simplex 1, Epstein Barr virus and Cytomegalovirus [51]. Consequently, an increasing number of clinical trials are focusing on the therapeutic value of miRNAs in viral infections. There are also several active clinical trials investigating miRNAs in other human diseases including in autoimmunity, various cancers and cardiovascular disease ([52], and at clinicaltrials.gov).

### 1.3. miRNAs and Bacteria

The vast majority of studies concerning miRNAs have focused on the role of miRNAs in eukaryotes, or in viruses that exploit eukaryotic cell machinery [7,51,52]. However, an expanding area of research is now showing that miRNAs can alter the composition of the bacteria in the intestinal microbiota of mammalian hosts [14,16]. Dysbiosis of the intestinal microbiota has been demonstrated in an increasing number of diseases ranging from Inflammatory Bowel Disease and Multiple Sclerosis, to psychological disorders including Bipolar Disorder, Depression and Anxiety amongst many others [53,54,55,56]. The mechanisms by which these unfavourable changes occur are largely unclear. Whether miRNAs could play a role in interactions between the more distantly related eukaryotes and bacteria is an emerging concept that requires more thorough consideration.

## 2. Regulatory RNAs in Bacteria

Bacteria possess three key classes of regulatory molecules—(i) *cis*-acting 5′ element non-coding RNAs, (ii) *trans*-acting small non-coding asRNAs (*trans*-asRNAs), and (iii) *cis*-encoded antisense RNAs (*cis*-asRNAs). *Cis*-acting 5′ element non-coding RNAs are typically present in the 5′UTR of the mRNA they regulate. Ligand binding induces structural changes in the non-coding RNA, that subsequently influences the transcription of the downstream gene. This type of RNA-mediated regulation includes riboswitches, thermoregulators and pH sensors [57]. Whereas, the bacterial *trans*-asRNAs and *cis*-asRNAs recognise their target sequence via complementary base pairing, akin to eukaryotic miRNAs, siRNAs and piRNAs. Eukaryotes and prokaryotes have utilised RNA-dependent mechanisms for various common functions including post-transcriptional gene regulation, as a defence against exogenous viral infections and as a defence against transposons (Table 1).

### 2.1. Bacterial Regulatory RNAs

Bacteria produce various classes of RNAs that can function as post-transcriptional gene regulators and can mediate protection against transposons and exogenous viruses [57,58]. The bacterial asRNAs regulate several critical processes including plasmid replication, conjugation, detoxification, quorum sensing, defence from transposable elements and the stress response [58,59,60,61,62]. A range of mechanisms are involved in RNA-mediated regulation of gene expression including transcription interference through the occlusion of promoters, degradation of sense transcripts, stabilisation of sense transcripts and regulation of translation. Overall, RNA-mediated repression of gene expression by *cis* and *trans-*asRNAs in bacteria is not well understood, in part due to the diverse mechanisms of action.

### 2.2. Cis-Acting asRNAs

*Cis*-asRNAs are transcribed from the strand that is antisense to the gene that they regulate, and therefore harbour overlapping complementary regions to the target gene [63,64]. Many *cis-*asRNAs are involved in the regulation of mobile genetic elements such as plasmids, phages, and transposons [65]. Other *cis*-asRNAs regulate responses to environmental changes and are critical for bacterial fitness [66]. *Cis*-asRNAs are typically short and require perfect complementarity to their target [65].

### 2.3. Trans-Acting asRNAs

*Trans*-asRNAs regulate genes located elsewhere on the chromosome. Bacterial *trans-*asRNAs also include the *trans*-acting CRISPR RNAs (tracrRNAs) that function in conjunction with CRISPR RNAs (crRNAs) in defence against bacteriophage [67]. *Trans-*asRNAs in bacteria share many common functions with miRNAs in eukaryotes. Furthermore, they have similar recognition mechanisms, in that they only require partial complementarity to their target sequence [57,64,68]. Less is known about the precise mechanisms of regulatory RNAs in bacteria, and mechanisms appear to vary considerably across different species due to the lack of conservation of key proteins. This is particularly evident when comparing the mechanisms in Gram-positive and Gram-negative bacteria.

### 2.4. asRNA-Mediated Regulation in Gram-Positive and Gram-Negative Bacteria

Interactions between asRNAs and their target mRNA can result in either degradation, or the protection from nuclease recognition and increased mRNA stability [69,70,71,72,73]. In Gram-negative bacteria, this nuclease is thought to be primarily RNase E, the key nuclease of the degradosome, in which RNase E acts as a scaffold for the binding of other enzymes including the RNA helicase RhlB, and the 3′–5′-exoribonuclease polynucleotide phosphorylase (PNPase) [74]. In Gram-positive bacteria, RNase Y is the key nuclease of the degradosome and is thought to be the primary nuclease of asRNA-mediated mRNA cleavage [74,75]. For many *trans*-asRNAs, the interaction with RNase E is mediated by the RNA chaperone protein, Hfq (Figure 1B) [29,30]. Hfq stabilises the *trans*-asRNA and facilitates binding between the *trans*-asRNA and its mRNA target, despite the limited complementarity in RNA sequence [72,76]. It also serves to protect many RNAs from degradation [77,78]. Hfq may also enable RNA export—due to its RNA binding properties and its ability to associate with, and create holes in, the lipid bilayers of liposomes and vesicles [79]. Hfq is widely conserved amongst bacteria, with around half of Gram-positive and Gram-negative bacteria expressing the protein [80]. In Gram-positive bacteria that do not possess RNase E, the role of Hfq is less clear, although in *Listeria monocytogenes* it has been shown to protect its target from nuclease degradation [81]. Hfq is also a recognised virulence factor in some Gram-negative bacteria including *Salmonella typhimurium* and *Vibrio cholerae* [82,83,84]. In addition to RNase E and RNase Y, RNase III is also involved in asRNA-mediated mRNA degradation, and RNase III is conserved amongst all bacteria due to its role in 16S and 23S ribosomal RNA (rRNA) maturation [66,72,85].

### 2.5. Trans-asRNAs in CRISPR

The tracrRNA of CRISPR, a bacterial defence against bacteriophage and invasive nucleic acids is also an example of a *trans*-asRNA. Here, the tracrRNA and the crRNA together form the guide RNA that directs the enzymatic action of the CRISPR-associated (cas) endonuclease to the target sequence [86]. The target nucleic acid sequence is that of an invasive bacteriophage or plasmid and is complementary to the spacer regions found within the crRNA. This enables sequence-specific recognition and degradation of foreign DNA. In type II CRISPR-Cas systems, for the arrays that contain multiple spacers the tracrRNA directs RNase III to the crRNA leading to its maturation [87].

## 3. Shared Features of Eukaryotic and Bacterial Regulatory RNA Pathways

### 3.1. A Conserved Protein Fold between AGO, PIWI and Cas

The viral-derived RNase H-like protein superfamily is thought to have been a key driver of the evolution of complex life forms on the early earth, as it includes many essential enzymes in both eukaryotes and bacteria [88]. Members of the RNase H-like protein superfamily are mediators of many important processes including DNA replication, transposition, gene splicing, and the generation of adaptive immune responses [89]. The RNA interference (RNAi) pathways of eukaryotes and the CRISPR anti-viral defence of bacteria both rely on RNase H-like endonucleases. In RNAi, this is the Argonaute and PIWI-like proteins, and in CRISPR, the Cas proteins [89]. These enzymes all possess an RNase H fold, one of the most ancient and abundant protein folds known [90]. At a glance, these distinct pathways and mechanisms can initially appear to be completely unrelated. However, there are many shared features of these pathways.

### 3.2. CRISPR RNA and piRNA-Guided Immunity

One of the most striking similarities between eukaryotic and prokaryotic regulatory RNA pathways is the crRNA-guided CRISPR immunity of prokaryotes and piRNA-guided immunity in metazoans. The piRNAs and crRNAs of these pathways each represent a form of genome-encoded trans-generational adaptive immunity. In prokaryotes, CRISPR mediates defence against invasive nucleic acids from bacteriophage and plasmids [67]. In metazoans, piRNA-mediated immunity is the primary defence mechanism against transposable elements in the germline [91]. The majority of piRNAs are derived from piRNA clusters [92]. Similarly, crRNAs are also derived from distinct regions in the prokaryotic genome, the CRISPR loci [67]. In prokaryotes, short sequences of the invasive DNA are integrated into the CRISPR loci by the Cas endonuclease, separated by repeat sequences. In eukaryotes, both invasive RNA and DNA viruses can be integrated into piRNA clusters [93,94]. Both piRNAs and crRNAs are classes of regulatory RNAs that are complementary in sequence to the invasive nucleic acid, and that guide an endonuclease complex to the foreign target sequence [94]. In CRISPR, this is the Cas endonuclease that is guided by crRNAs, whereas piRNAs guide the PIWI endonuclease to the target sequence. In contrast to CRISPR, piRNAs can also direct histone modifications that mediate gene silencing [95]. These pathways are prime examples of the parallel functions and mechanisms of the distinct regulatory RNA pathways in eukaryotes and bacteria.

### 3.3. Shared Features of mRNA Decay in Eukaryotes and Bacteria

In eukaryotes, newly synthesised mRNAs are characterised by a 5′ 7-methylguanylate (m^7^G) cap and 3′ poly-adenylation [96]. General mRNA decay occurs due to decapping of the mRNAs and removal of the poly (A) tails in cytoplasmic Processing bodies (P-bodies) [97]. In bacteria, the degradosome is the key mediator of bulk RNA turnover, and newly synthesised bacterial mRNAs possess a 5′ triphosphate group that must be converted to a 5′ monophosphate group for efficient recognition by RNase E or RNase Y of the degradosome [98]. Interestingly, both miRNAs and siRNAs in eukaryotes are also characterised by 5′ monophosphate groups, and the enzyme responsible for this conversion in *E. coli*, RppH, is closely related to DCP2 of the decapping complex in eukaryotes [99,100,101]. The DCP2 decapping complex is also known to be involved in miRNA-mediated gene silencing in eukaryotes [102]. The 5′ monophosphate may enable the association of miRNAs and siRNAs with the nucleases of the bacterial degradosomes. Whether this shared evolutionary relationship between decapping enzymes could also enable miRNA-mediated regulation of bacterial gene expression through the degradosome, is another interesting prospect. A further example of this shared relationship between eukaryotic and bacterial RNA decay pathways is also demonstrated by Hfq of the *trans*-asRNA pathway, and its likeness to the eukaryotic Sm proteins.

### 3.4. Bacterial Hfq is Reminiscent of Eukaryotic Decapping Proteins

The function of Hfq is somewhat analogous to the RISC complex, as it facilitates interactions between *trans*-asRNAs and their target mRNAs [103]. Yet, it bears the most striking structural and functional similarities to the eukaryotic Sm and Sm-like proteins [104,105]. It contains the conserved Sm1 motif and, similar to the eukaryotic proteins, forms an oligomeric ring-shaped structure. Hfq facilitates the regulation of mRNA stability through interactions with *trans*-asRNAs, whereas in eukaryotes the Sm-like protein, Lsm1, is involved in mRNA decapping and degradation [106]. These conserved structural and functional components may enable *trans*-asRNAs of bacteria to interact with the mRNA decay machinery in eukaryotes. Alternatively, they may enable miRNAs that function through decapping enzymes to influence bacterial gene expression. Investigating the ability of eukaryotic regulatory RNAs to complex with Hfq or the bacterial degradosome, and of *trans*-asRNAs to interact with eukaryotic Sm-like proteins could reveal novel mechanisms of host-bacterial interactions.

## 4. miRNAs Facilitate Inter-Kingdom Communications

There are several examples of inter-kingdom communications mediated by miRNAs and miRNA-like molecules amongst the eukaryotes. The processes of RNA release and the mechanisms by which they remain resistant to degradation in the environment are critical to RNA-mediated inter-kingdom interactions. Extracellular Vesicles (EVs) containing RNA as cargo offer a route by which this communication could be mediated.

### 4.1. EVs in Eukaryotes and Bacteria

EVs are spherical membranous structures that are shed from the surface of cells. There are three main types of EVs in eukaryotes: exosomes, apoptotic blebs and shedding microvesicles, that vary in size and biogenesis [107]. The production of EVs is conserved across the domains of Archaea, Bacteria and Eukarya. In bacteria, these are sometimes referred to as Outer Membrane Vesicles (OMVs), Outer-Inner Membrane Vesicles (O-IMVs), or Membrane Vesicles (MVs) but will be referred to collectively as EVs from herein [108]. In both eukaryotes and prokaryotes, EVs appear to enable the shuttling of genetic information between cells, facilitating horizontal gene transfer (HGT) in bacteria and long-distance DNA and RNA transfer in eukaryotes [109,110,111]. Bacteria are known to export a range of RNAs in EVs including rRNA, transfer RNA (tRNA), transfer-messenger RNA (tmRNA), mRNA and most notably, some bacteria have even been shown to export *trans*-asRNAs, and microRNA-size RNAs (msRNAs) in EVs [33,34,35]. Many of the small RNAs found in bacterial EVs are often enriched compared to the cytoplasm of the cell that they are derived from [112,113]. Helminth parasites such as *Schistosoma japonicum*, *Heligmosomoides bakeri* and *Trichuris muris* also produce EVs containing RNA that have important immunomodulatory effects in the host [114,115,116]. Whilst, EVs from plants have been shown to enable the transfer of functional miRNAs from plants to mammals, representing a means by which plant substances in the human diet can directly regulate mammalian gene expression and immune function [117].

### 4.2. Plant miRNAs in Inter-Kingdom Communications

During initial studies, it was demonstrated that rice-derived miR-168a is stably present in human serum after a rice meal and that this can target human/mouse low-density lipoprotein receptor adapter protein 1 (LDLRAP1) mRNA, decreasing its expression in the liver [117]. Similar studies have detected other dietary-derived plant miRNAs in various animal tissues [118,119]. However, these studies remain controversial amid claims that these findings were due to sample contamination, and that the quantity detected was not biologically significant. Other studies have subsequently counteracted these criticisms (reviewed in [120]).

Supporting studies have detected plant-derived Exosome-like nanoparticles (ELNs) in grape, grapefruit, carrot and ginger [121]. These ELNs are resistant to degradation by acidic stomach-like solutions and were found to contain miRNAs; some of which share a seed sequence with known human miRNAs. As such, they offer a potential mechanism for the transport of functional plant-derived RNA molecules into heterologous hosts following consumption. However, further work is needed to confirm this concept. Plant siRNAs and miRNAs also serve important roles in plant immune responses, and influence interactions with various pathogens including fungi, viruses, bacteria and helminths (reviewed in [122]).

### 4.3. Human and Plant miRNAs in EVs Can Modulate the Gut Microbiota

Bacteria have been overlooked as potential targets for eukaryote-derived miRNAs, given their lack of known machinery necessary for the production and function of these molecules in eukaryotes. However, recent work has challenged this concept [14]. Their studies showed that host Intestinal Epithelial Cells (IECs) produced miRNA-containing EVs under healthy physiological conditions. Furthermore, these EVs were internalised by specific members of the intestinal microbiota, and the miRNAs within them found to co-localise with their nucleic acids and alter their growth [14]. Plant ELNs containing miRNAs are also internalised by specific members of the intestinal microbiota [16]. Ginger ELNs (GELNs) contain 109 mature miRNA sequences, which can increase the levels of Lactobacillaceae and inhibit the growth of Ruminococcaceae. In these studies, treatment of the probiotic bacterium, *Lactobacillus rhamnosus* (LGG) with GELNs led to reduced expression of 249 mRNAs. One of the GELN miRNAs, mdo-miR7267-3p was shown to target the LGG monooxygenase, YcnE, and alters tryptophan metabolism by increasing the production of Indole-3-carboxaldehyde (I3A) from tryptophan [16]. I3A is a ligand for the mouse aryl hydrocarbon receptor (AHR) and was sufficient to induce the production of IL-22, leading to an improvement in intestinal barrier function and an enhanced resistance to DSS-induced colitis [16]. This demonstrates the role of eukaryotic miRNAs in the regulation of the mammalian microbiota.

Furthermore, the periodontal pathogen *Porphymonas gingivalis* internalises GELNS through binding to a surface protein, hemin-binding protein 35 (HBP35). GELNs decrease the ability of *P. gingivalis* to attach, enter and proliferate in oral epithelial cells. Whilst, a GELN miRNA, aly-miR159a was shown to significantly reduce the expression of the gingipain toxin genes Rgp and Kgp [15]. This study signifies that plant miRNAs from the diet can potentially influence the disease outcome of a significant human pathogen, whilst offering a potential novel avenue by which the human microbiota can be modulated.

### 4.4. The Microbiota Can Modulate Host miRNA Expression

The interaction between the microbiota and miRNAs is a bi-directional relationship. Studies comparing the miRNA profiles of germ-free (GF) mice and conventionally raised mice show that the microbiota negatively regulates the expression of miR-10a by intestinal dendritic cells, in a TLR-TLR and MYD88-dependent pathway [123]. This in turn reduces the production of IL-12, an inducer of IFN-γ and a driver of the Th1-type inflammatory immune responses. Consequently, limiting intestinal inflammation and improving barrier function under normal physiological conditions [123]. The microbiota also modulates the host miRNA profile through the production of bioactive metabolites. Polyphenols are found in plant products including seasonings, nuts and berries, and can be metabolised into bioactive urolithins by the gut microbiota. One such compound, urolithin A, induces an increase in miR-10a-5p expression and inhibits the activation and proliferation of CD4+ T cells through the down-regulation of Orai1 and STIM1/2 transcripts [124]. This mechanism may confer the anti-oxidative and anti-inflammatory effects shown by these foods. It is clear that the bacterial modulation of the host miRNA profile can occur through various distinct mechanisms, and that this is important in host-microbiota interactions.

## 5. Mechanisms for RNA-based Communication between Eukaryotes and Bacteria

### 5.1. Bacterial asRNAs Can Hijack Eukaryotic RNAi Pathways

Several studies have illustrated the presence of msRNAs in bacterial genomes using bioinformatic approaches over the last decade [125,126,127]. These bacterial-derived msRNAs can be delivered into host cells and incorporate into the eukaryotic host’s miRNA-processing machinery, thereby serving important functional roles for the bacterium [47,128]. For example, an msRNA derived from an RNA stem-loop in *Mycobacterium marinum* associates with eukaryotic RISC [128]. Furthermore, *Salmonella* can release RNA into the cytosol of infected IECs, and this RNA is processed by human AGO2, in a Dicer-independent fashion, into small functional msRNAs of 22 nucleotides in length. One of these miRNA-like molecules, Sal-1, serves as a critical virulence factor, facilitating the intracellular replication and survival of the bacteria [47]. These data demonstrate a role for bacterial-derived RNA molecules in the successful colonisation by human pathogens.

Similarly, it was observed that the periodontal pathogens *Aggregatibacter actinomycetemcomitans*, *P. gingivalis*, and *Treponema denticola* produce msRNAs in EVs that could enter eukaryotic fibroblast cells in vitro [129]. Exogenous transfection of some of the most abundantly expressed msRNAs by these bacteria led to a decrease in the anti-inflammatory cytokines IL-5 and IL-13 by Jurkat T cells [129]. These pathogens have been implicated in periodontitis, which is driven by the inflammatory response to the bacteria of the oral microbiota [130]. This work, therefore, highlights bacterial RNAs as a potential novel therapeutic target.

Additionally, *trans*-acting asRNAs OxyS and DsrA from *E. coli*, can impair *C. elegans* chemosensory behaviour and longevity through down-regulation of *che-2* and the diacylglycerol lipase gene *F42G9.6*, respectively [35]. This is an RDE-4 dependent process, with RDE-4 being a key component of *C*. *elegans* RNAi, that processes dsRNA by complexing with Dicer and subsequently worm argonaute, RDE-1 [35,131]. In *E. coli* OxyS and DsrA function to co-ordinate the stress response through the regulation of the protein sigma factor RpoS in an Hfq-dependent manner [132]. Together, these studies show that a number of bacteria are known to produce RNAs that could mimic eukaryotic regulatory RNAs and feed into eukaryotic RNAi processing pathways. In addition, bacterial asRNAs can also have additional distinct functions in the eukaryotic host. Whether these relationships extend to host-microbiota interactions is an intriguing prospect that warrants further study. Investigating whether the production of RNA by members of the microbiota can modulate relationships with the host, and vice versa will be a key part of translating correlative microbiota studies into clinical benefits.

### 5.2. Proposal of Mechanisms for Regulatory RNAs in Inter-domain Communications

**Figure 2:**
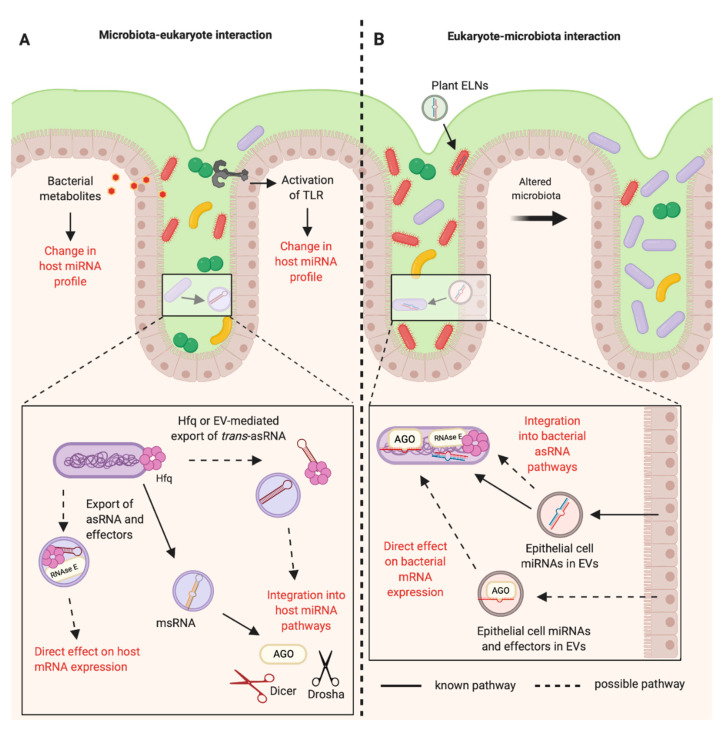
RNA-mediated communications between the mammalian host and the bacteria of the gastrointestinal microbiota. (**A**) The bacteria of the mammalian intestinal microbiota can indirectly alter the host miRNA profile through the production of metabolites and activation of Toll-like Receptors (TLRs). It is known that bacteria can produce microRNA-size RNAs (msRNAs) in Extracellular Vesicles (EVs) that can integrate into host miRNA pathways [47]. Other RNAs such as *trans-*asRNAs could be exported in vesicles and could similarly integrate into host pathways. It is also possible that asRNAs could be exported along with their effectors in EVs (such as Hfq and RNase E) and could directly influence host gene expression. EV-independent export of asRNAs could be mediated by Hfq, as Hfq can create holes in the bacterial cell membrane [79]. **(B**) Plant exosome-like nanoparticles (ELNs) from diet and EVs from intestinal epithelial cells containing miRNAs can enter members of the microbiota and influence bacterial gene expression and growth [14,16]. These miRNAs could be exported with effectors such as Argonaute (AGO) to directly alter bacterial gene expression. Alternatively, eukaryotic miRNAs could become functional upon integration into existing bacterial asRNA regulatory pathways (Created with BioRender.com).

This review of the current literature leads us to propose two potential mechanisms by which regulatory RNAs could mediate bi-directional communications between eukaryotes and bacteria. (1) RNA molecules produced by one organism could integrate into one of the distinct regulatory RNA pathways present in the other. For example, the RNAs produced by *Salmonella* become functional upon processing by eukaryotic RNAi machinery, such as AGO2 and Dicer [47]. This is also true for *E. coli*, which exploits *C. elegans* RNAi machinery to regulate eukaryotic gene expression through its own bacterial *trans*-asRNAs [35] (2) RNAs are exported in EVs in conjunction with the molecules required for their functioning in the recipient (Figure 2). This is shown by Hfq which is exported in EVs by *Yersinia pestis*, and in an *Acinetobacter baumannii* Hfq mutant which displays reduced EV production [36,133]. Furthermore, eukaryotic Argonaute has also been detected in EVs [134]. These mechanisms could also occur in combination, with both host and recipient molecules important in inter-domain regulatory RNA-mediated communications.

## 6. Future Directions

Development of a robust miRNA-mediated bacterial phenotype in a genetically tractable bacterial species in vitro will enable the interrogation of the mechanisms of action. Researchers could seek to knock-out or inhibit the expression of key molecules such as bacterial Hfq, to determine whether these molecules are required for miRNA function in the bacterial recipient. Further interrogation of the contents of EVs containing miRNAs in eukaryotes could also yield insights into their functions in bacteria. Furthermore, we note that many diseases are associated with perturbations to both the host miRNA profile and host microbiotas. This is evident in cancers including Colorectal cancer, Ovarian cancer and Hepatic carcinoma, and among a wide range of other diseases including Autistic Spectrum Disorder, Crohn’s Disease and Multiple Sclerosis [135,136,137,138]. Future investigations could seek to determine if miRNAs are responsible for the microbiota perturbations in these contexts. We anticipate that this approach could establish causality in several correlative studies.

## 7. Concluding Remarks

Eukaryotic regulatory RNAs have recently been implicated as regulators of bacterial growth. However, due to the lack of proposed mechanisms for these interactions these studies remain controversial. Here, we sample the significant body of research illustrating the role of regulatory RNAs in various inter-kingdom communications. From human-pathogen interactions and human-microbiota interactions to diet-microbiota interactions and the health implications of dietary-derived miRNAs, this research is significant in both medicine and agriculture. Amongst eukaryotes, it is clear that regulatory RNA pathways are largely conserved between animals and plants. However, there are significant gaps in the literature with regards to the molecular mechanisms of the proposed interactions between eukaryotes and bacteria. We anticipate that our careful comparisons of the molecular pathways of RNA-mediated gene regulation in eukaryotes and bacteria could significantly aid developments in the field of inter-domain communications. As EVs and regulatory RNAs are produced either directly or indirectly by all forms of life, we propose that RNA encapsulated in EVs could enable communication between all organisms, even the distantly related eukaryotes and bacteria. Future studies should seek to investigate whether eukaryotic miRNAs that influence bacterial growth could feed into the RNA processing pathways of bacteria, or whether they are exported with eukaryotic RNAi machinery. This will be particularly informative for the treatment of microbiota-associated diseases and bacterial pathogens and is essential for validating the concept of regulatory RNAs as a truly universal language.

## Authors Contribution

E.L. did the original draft preparation, writing and construction of figures, A.-M.F., S.G.-J., R.K.G. and I.S.R. were involved in writing, reviewing and editing the manuscript. All authors have read and agreed to the published version of the manuscript.

## Figures and Tables

**Table 1:** Illustrating the similarities between eukaryotic and bacterial antisense RNA-mediated regulatory pathways.

Eukaryotic RNA	Length	Functions	Mechanisms	Distribution	Bacterial RNA	Length	Functions	Mechanisms	Distribution
**miRNA**	21–25 nucleotides	Translational repression and transcriptional decay Defence against exogenous viruses Transposon defence Epigenetic regulation	Imperfect complementarity to target RNase III enzymes for biogenesis (Dicer and Drosha) Typically bind the 3’UTR of target mRNA AGO-dependent degradation and translational repression Chromatin modification through targeting epigenetic factors	Dicer and Drosha-independent mechanisms also exist Found in most eukaryotes	***Trans*** **-acting asRNA**	Typically long (100s–1000s of nucleotides)	Translational repression and transcriptional decay Defence against exogenous viruses Transposon defence	Imperfect complementarity to target Usually derived from intergenic regions Can target mRNA or protein Hfq as a chaperone RNase E, RNase Y or RNase III-mediated cleavage	Hfq is only present in some bacteria RNase E is only present in Gram-negative bacteria RNase Y is only present in Gram-positive bacteria
**siRNA**	22–25 nucleotides	Translational repression and decay Defence against exogenous viruses Transposon defence Epigenetic regulation	Perfect complementarity to target RNase III enzymes for biogenesis (Dicer) AGO-dependent degradation Histone modification	Found in most eukaryotes	***Cis*** **-acting asRNA**	Mostly short	Translational repression and transcriptional decay Transposon defence Regulation of plasmid copy number, conjugation and phage life cycle Environment-mediated regulation of gene expression	Perfect complementarity to target Located opposite the mRNA it regulates Can be structural inhibition of transcription/translation RNase E, RNase Y or RNase III-mediated cleavage	RNase E is only present in Gram-negative bacteria RNase Y is only present in Gram-positive bacteria
**piRNA**	24–31 nucleotides	Transposon defence in germline Transgenerational epigenetic inheritance Expressed in somatic cells and bodily fluids where role is unclear	Perfect complementarity to target for canonical piRNAs Imperfect complementarity to target for other piRNAs Often derived from piRNA clusters PIWI-dependent degradation	Found in metazoans Biogenesis is highly variable amongst different organisms	**crRNA** **tracrRNA**	20 nucleotide spacers in crRNA Approx. 100 nucleotides in tracrRNA	Defence against invasive bacteriophage and plasmids	crRNAs have perfect complementarity to target tracrRNAs have imperfect complementarity Derived from CRISPR loci Cas9-dependent degradation	Found in 50% of bacteria 90% of archaea
